# Bio-Based Plasticized PVA Based Polymer Blend Electrolytes for Energy Storage EDLC Devices: Ion Transport Parameters and Electrochemical Properties

**DOI:** 10.3390/ma14081994

**Published:** 2021-04-16

**Authors:** Shujahadeen B. Aziz, Muaffaq M. Nofal, M. F. Z. Kadir, Elham M. A. Dannoun, Mohamad A. Brza, Jihad M. Hadi, Ranjdar M. Abdullah

**Affiliations:** 1 Hameed Majid Advanced Polymeric Materials Research Lab., Physics Department, College of Science, University of Sulaimani, Qlyasan Street, Sulaimani 46001, Iraq; ranjdar.abdullah@univsul.edu.iq; 2 Department of Civil Engineering, College of Engineering, Komar University of Science and Technology, Sulaimani 46001, Iraq; 3 Department of Mathematics and General Sciences, Prince Sultan University, P.O. Box 66833, Riyadh 11586, Saudi Arabia; muaffaqnofal@gmail.com; 4 Centre for Foundation Studies in Science, University of Malaya, Kuala Lumpur 50603, Malaysia; mfzkadir@um.edu.my; 5 Associate Director of General Science Department, Woman Campus, Prince Sultan University, P.O. Box 66833, Riyadh 11586, Saudi Arabia; elhamdannoun1977@gmail.com; 6 Department of Manufacturing and Materials Engineering, Faculty of Engineering, International Islamic University of Malaysia, Kuala Lumpur 53100, Malaysia; mohamad.brza@gmail.com; 7 Department of Medical Laboratory of Science, College of Health Sciences, University of Human Development, Sulaimani 46001, Iraq; jihad.chemist@gmail.com

**Keywords:** PVA, methylcellulose, glycerol plasticizer, circuit modeling, ion transport parameters, electrochemical properties, EDLC fabrication

## Abstract

This report shows a simple solution cast methodology to prepare plasticized polyvinyl alcohol (PVA)/methylcellulose (MC)-ammonium iodide (NH_4_I) electrolyte at room temperature. The maximum conducting membrane has a conductivity of 3.21 × 10^−3^ S/cm. It is shown that the number density, mobility and diffusion coefficient of ions are enhanced by increasing the glycerol. A number of electric and electrochemical properties of the electrolyte—impedance, dielectric properties, transference numbers, potential window, energy density, specific capacitance (*C_s_*) and power density—were determined. From the determined electric and electrochemical properties, it is shown that PVA: MC-NH_4_I proton conducting polymer electrolyte (PE) is adequate for utilization in energy storage device (ESD). The decrease of charge transfer resistance with increasing plasticizer was observed from Bode plot. The analysis of dielectric properties has indicated that the plasticizer is a novel approach to increase the number of charge carriers. The electron and ion transference numbers were found. From the linear sweep voltammetry (LSV) response, the breakdown voltage of the electrolyte is determined. From Galvanostatic charge-discharge (GCD) measurement, the calculated *C_s_* values are found to drop with increasing the number of cycles. The increment of internal resistance is shown by equivalent series resistance (*ESR*) plot. The energy and power density were studied over 250 cycles that results to the value of 5.38–3.59 Wh/kg and 757.58–347.22 W/kg, respectively.

## 1. Introduction

The field of polymer electrolytes (PEs) is extended from polymer science to a number of areas, including electrochemistry organic chemistry and even inorganic chemistry. Rapid change in human lifestyle demands high performance energy storage devices (ESDs) [1,2]. The conducting PEs were greatly developed in this era due to their promising performance for the ESDs applications such as electrochemical double layer capacitor (EDLC) and proton batteries. The mentioned applications have been recognized as promising replacements to conventional batteries because of their cost effective and simple fabrication method with a satisfactory performance, which is able to achieve good values of power density and specific capacitance [1,2,3]. The most used electrode material for the construction of EDLC is activated carbon. Earlier studies have proven that the activated carbon electrode with large and porous surface area causes an increase of conductivity and other important EDLC parameters such as energy and power density [4]. A non-Faradaic mechanism of energy storage (ES) is used in the EDLC where a double layer (DL) at the area of the electrolyte-electrode is created by the ions transfer or accumulation of charges where there is no involvement of electrons [5]. The usage of solid polymer electrolyte (SPE) in these applications has also been acknowledged by few researchers because of their ability to provide good mechanical stability and good electrolyte/electrode contact [6]. SPEs also have much less tendency to harm the internal components of the devices because they circumvent leaking and corrosive problems that are possessed by liquid electrolyte [7,8,9]. Verma et al. [10] prepared nano-composite PE of 95(70PEO-30AgI)-5SiO_2_ with the conductivity value of 2.5 × 10^−3^ S/cm. They showed that the electrolyte is appropriate for applications in solid capacitor owing to the conductivity improvement and enhancement in amorphous phase. The preparation of process of SPEs is also straightforward and the electrolyte itself is long lasting [11]. The blend of PEs is shown to create an exceptional electrolyte film with good thermal and chemical durability that appropriate to be applied in a particular application [12]. This is because the technique of blending has confirmed to enrich the vacant sites for ions to hop through the PEs therefore, improve the conductivity of the film [13,14].

The polyvinyl alcohol (PVA) polymer has been widely utilized in conducting PE in two forms: pure polymer [15,16,17] and blended polymer including polyvinyl pyrrolidone [18], arginine [19] and carboxymethyl cellulose [20]. For reformulation of PEs, PVA is one of appropriate polymers because of several advantageous properties, including semi-crystalline, non-toxicity, satisfactory strength and sufficient charge storing capability [21,22]. It is also worth-mentioning that PVA contains hydroxyl group (-OH) in the methyl carbon which forms hydrogen bonding [16]. Similarly, methyl cellulose (MC) as a derivative of cellulose is an alternative of PVA. The methyl cellulose (MC) consists of β-(1,4)-glycosidic bond attached to methyl substituents in linear chains [23] that is capable of providing polarity via oxygen atoms that contain lone pair electrons [24]. It is also of great importance to know that MC possesses amphiphile property originating from hydrophobic polysaccharide and hydrophilic carboxylic functional group [25].

The usage of single MC-based electrolytes in this field has been documented [26,27,28]. It has also been used by mixing with potato starch [29], maize starch [30] and chitosan [31]. This is due to unique properties of MC, such as non-toxicity, biocompatibility, mechanical strengthens and thermal stability [32]. The ionic conductivity of an electrolyte has to be determined and sufficient to be used in energy devices. This property can be enhanced via either salts and/or plasticizers additions [33,34]. The small size ion, such as Li^+^ ion has widely been utilized; however, it is harmful to environment because of non-biodegradability and expensiveness [35].

To solve this issue, ammonium-based salts, for instance ammonium iodide (NH_4_I) has replaced lithium salt effectively by providing cation and anion to PE composites [36]. For instance, NH_4_I has a low lattice energy of 605.3 kJ/mol, indicating a high degree of salt dissociation into ions [37]. Moreover, ammonium-based salts are commonly used in the formulation of PE system because they could accomplish high ionic conductivity with good compatibility and thermal stability [38]. Additionally, the addition of plasticizers into PE could also modify the basic interaction of polymers and affects the ionic conductivity of the system [39]. Various types of plasticizers have been reported in other works such as polyethylene glycol [24], propylene carbonate [40] and ethylene carbonate [41]. Most of the PE that would be fabricated in the energy devices is formulated by adding glycerol to enhance the ionic conductivity as well as other important parameters [42,43]. Furthermore, glycerol has been proven to increase the ionic conductivity up to ~10^−3^ S/cm, by creating more pathways for ions conduction hence revealing an adequate achievement in EDLC application, where the energy and power densities were 3.1 Wh/kg and 910–385 W/kg, respectively, with cycle number of 1000 [44]. Additionally, glycerol reduces the electrostatic force amongst cations and anions of the salt and thus produce more mobile ions [44]. An electrolyte film and two electrodes’ materials are inserted in the EDLC device. Generally, supercapacitors (SCs) are made using carbon-based electrodes [45]. The various carbon-based electrodes used in EDLCs are carbon nano-tubes, carbon nano-fibers, carbon aerogels, activated carbon (AC), graphites and carbon nano-size [46]. The electrode material is used in this work is AC owing to its high porosity with more than 2 nm in pore width, high conductivity, large surface area with more than 1000 m^2^ g^−1^, cost effectiveness and chemical stability [47,48]. AC has high surface area of 2500 m^2^/g compared with the carbon black (CB). More ions could be adsorbed due to high surface area of AC and hence the DL is synthesized because of the ion accumulation. This is why the AC is known as the active material. CB has a very small surface area and would be used to enhance the electrodes electrical conductivity [2]. This work reports the effect of PE that is formulated with different concentration of glycerol using several experimental techniques. The most favorable electrolyte with the highest conductivity value will be applied in the EDLC fabrication.

## 2. Experimental

### 2.1. Material and Preparation of Blend SPE Films

Methyl cellulose (MC) with the average molecular weight (Mw_avg_) of 4000 cP and polyvinyl alcohol (PVA) [purity 98%] with the Mw_avg_ of 35,000 g/mol, respectively, were used as raw materials and purchased by Sigma Aldrich (Kuala Lumpur, Malaysia). Ammonium iodide (NH_4_I) salt (Sigma Aldrich, Kuala Lumpur, Malaysia) was used to provide H^+^ ions in the electrolyte. The PVA:MC:NH_4_I electrolyte films are prepared by the raw materials using solution casting technique. For this purpose, 30 mL of distilled water was used to dissolve 20 wt.% of MC at room temperature for 6 h. At the same time, separately 80 wt.% of PVA was dissolved in 30 mL of distilled water at temperature of 80 °C. When the PVA solution was cooled to room temperature, PVA and MC solutions were blended using magnetic stirrer. Consequently, 40 wt.% of NH_4_I salt was added to the solution of PVA:MC blend and stirred continuously to get PVA:MC:NH_4_I electrolyte. To prepare plasticized PE 10 to 50 wt.% of glycerol (Sigma Aldrich, Kuala Lumpur, Malaysia) was incorporated to the PVA: MC: NH_4_I electrolyte separately. The electrolytes were coded as MCPVI1, MCPVI2, MCPVI3, MCPVI4 and MCPVI5 for the PVA:MC:NH_4_I incorporated with 10 wt.%, 20 wt.%, 30 wt.%, 40 wt.% and 50 wt.% of glycerol plasticizer, correspondingly. Finally, the electrolyte solutions were poured into different clean and dry plastic Petri dishes, subsequently left to evaporate gradually at ambient temperature, to attain a free-standing and dry of PVA:MC:NH_4_I blend SPE film.

### 2.2. EIS Measurements

The measurement of impedance of the series of PEs was performed via electrical impedance spectroscopy (EIS) using HIOKI 3532-50 LCR HiTESTER (HIOKI, Nagano, Japan) (50 Hz ≤ *f* ≤ 5 MHz) at ambient temperature. The impedance data was measured by inserting the PEs between two stainless steels. The analysis provided dielectric behavior and ionic conductivity of the electrolytes. The following relationship was used in calculating ionic conductivity:

(1) $$\sigma_{dc}=\left(\frac{1}{R_{b}}\right)\times \left(\frac{t}{A}\right)$$

where: *t* is the sample thickness, *A* is the electrode area and *R_b_* is the bulk resistance, which is determined from the intersection of the tail with real axis [49].

### 2.3. Linear Sweep Voltammetry (LSV) and Transference Number Measurement (TNM)

The analysis using a linear sweep voltammetry (LSV) study enabled us to determine the breakdown voltage of an electrolyte and this explained the system electrochemical stability. The LSV was performed at a scan rate of 10 mV/s using Digi-IVY DY2300 potentiostat (Neware, Shenzhen, China). Furthermore, the ions dominancy was determined by plotting the polarization of electrolytes against time. The plot was obtained through a V&A Instrument DP3003 (V & A Instrument, Shanghai, China) supported with a digital DC power supply. The measurement was done with operating voltage of 0.2 V at room temperature.

### 2.4. EDLC Preparation

**Figure 1:**
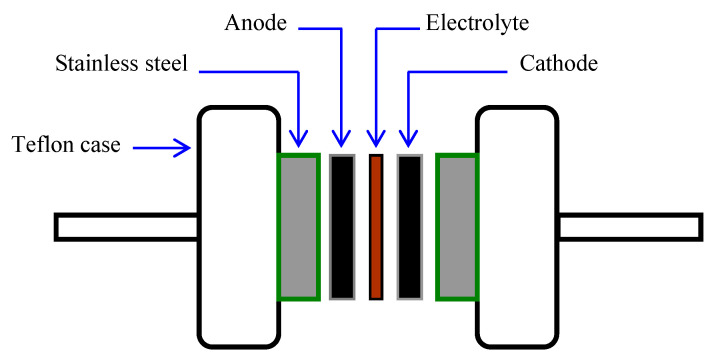
Diagram of the EDLC cell.

There were three primary materials to prepare the electrode in this study, which were activated carbon (Sigma Aldrich, Kuala Lumpur, Malaysia) as the active electrode material, CB as the electronic conductor and polyvinylidene fluoride (PVdF). 3.25 g of activated carbon was stirred together with 0.25 g of CB in a planetary ball miller (Neware, Shenzhen, China) at 500 rpm for ~20 min using six metal balls. Simultaneously, 0.5 g of PVdf was mixed in 15 mL of *N*-methyl pyrrolidone (NMP) solvent (Sigma Aldrich, Kuala Lumpur, Malaysia). The mixed powders from ball miller were blended with the PVdF/NMP solution for 2 h to obtain a black thick solution that was then poured onto an aluminum foil. It was dried in 60 °C oven for a certain time to form a carbon-based electrode with high conducting characteristics. The EDLC could be fabricated at this stage in a coin cell by using the electrolyte/electrode arrangement as reported in our previous works [50]. The Galvanostatic charge-discharge (GCD) measurement was used for the synthesized EDLC cell. A Neware battery cycler (Neware, Shenzhen, China) was utilized for this measurement at a current density (J) of 0.5 mA/cm^2^. The EDLC coin cell using the electrolyte/electrode arrangement is shown in Figure 1.

## 3. Results and Discussion

### 3.1. Impedance Study

**Figure 2:**
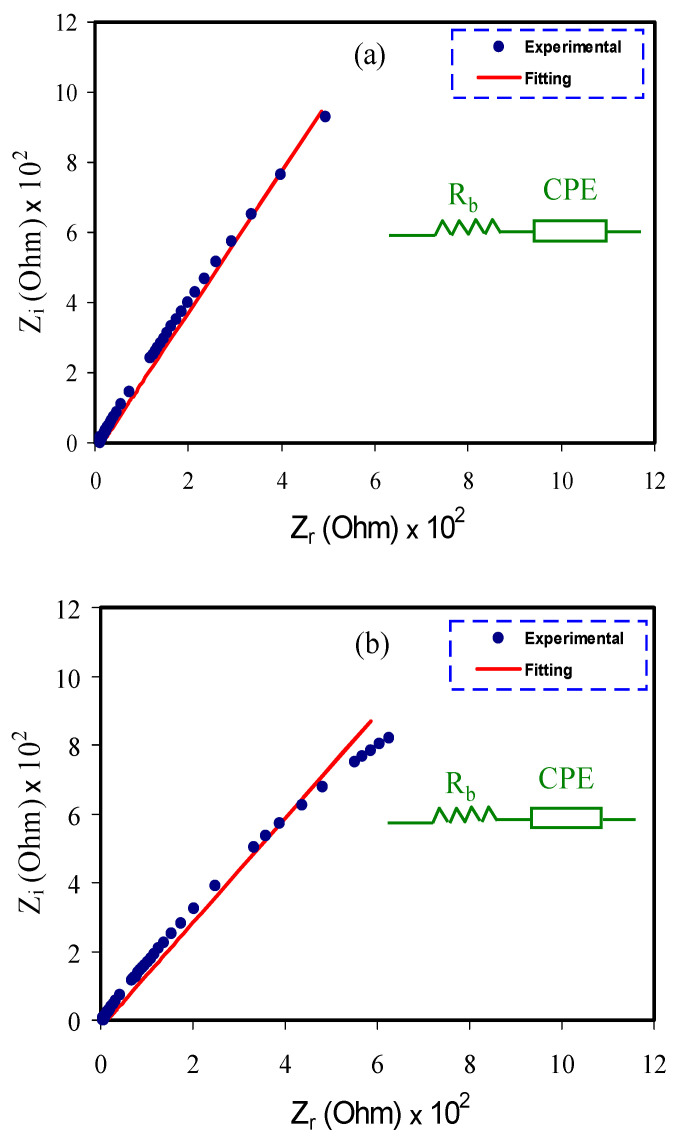

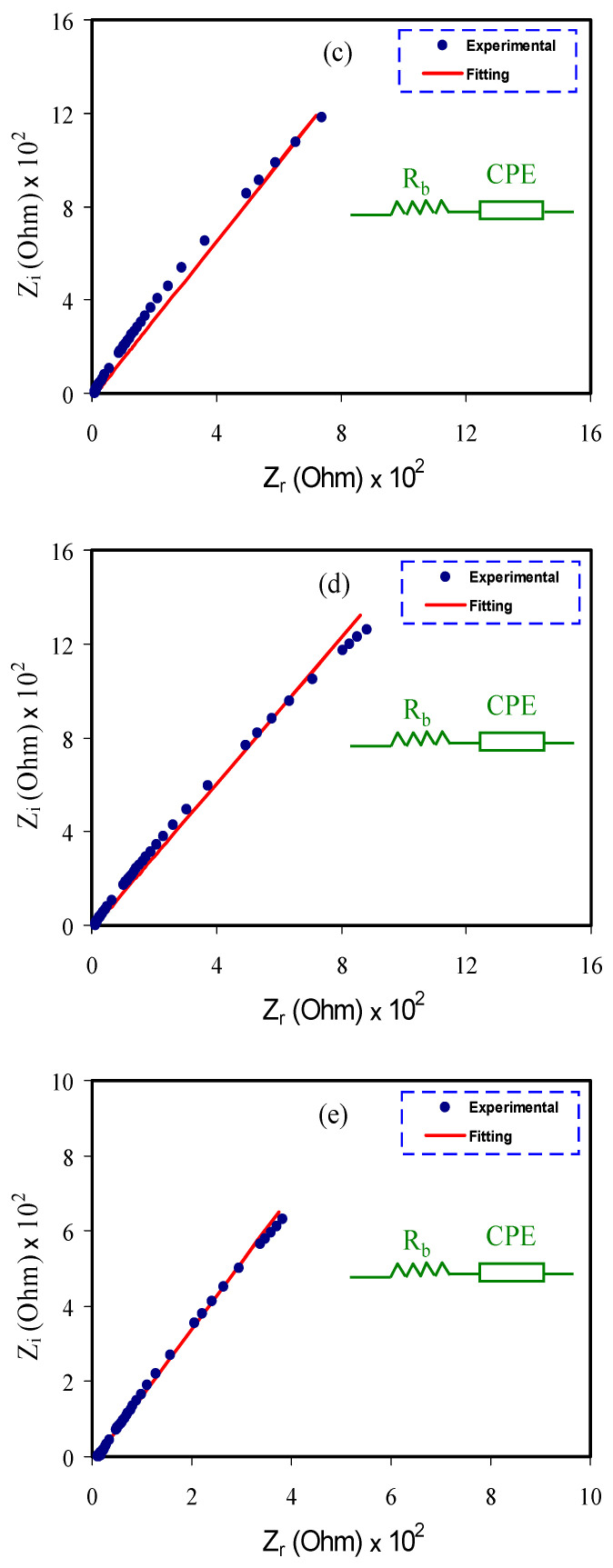
EIS plots for (**a**) MCPVI1, (**b**) MCPVI2, (**c**) MCPVI3, (**d**) MCPVI4 and (**e**) MCPVI5 electrolyte films.

To deal with charge transfer and ion transport, it was of great opportunity to use electric impedance spectroscopy (EIS) [51]. The Nyquist plots for all films are shown in Figure 2a–e. All plots were characterized by a tail at the low frequency region. The spike was related to creation of DL capacitance at the interfacial region [52,53]. At the low frequency region, it is assumed that a straight line is a parallel to the axis of imaginary, meaning that the straight line inclination must be 90° in the EIS plots. However, the inclination of the straight line at 90° resulted from the DL capacitance at the electrodes [52]. Semicircle at the high frequency region did not appeared in Figure 2a–e since most of the anions and cations at the bulk of the electrolyte (BE) transported in opposite direction, towards the stainless steel electrodes to generate the DL capacitor at the electrode-electrolyte interfaces. Different electrode materials were prepared in previous studies for SCs electrode application [54,55,56]. For example, Gomaa A. M. Ali et al. [57] synthesized MoS_2_:grapheme (MSG) composite as electrode material for SCs application. They used EIS to investigate the electrochemical behavior of the MSG composite. A semicircle and a linear portion at the high and low frequency region were observed, respectively. The authors showed that the MSG composite electrode had small values of solution resistance (R_S_) of 0.76 Ω·cm^2^ and charge transfer resistance (R_ct_) of 0.65 Ω·cm^2^.Gomaa A. M. Ali et al. [58] in another study prepared activated carbon from palm kernel shell (ACPKS) and impregnated with CaO from egg shell (CaO/ACPKS) as SCs electrodes. The Rs values were determined to be 2.62 and 0.92 Ω for ACPKS electrode and CaO/ACPKS electrode, respectively. The values of R_ct_ were determined to be 0.86 and 0.44 Ω for ACPKS electrode and CaO/ACPKS electrode, respectively.

Determination of the *R_b_* could be performed from the intercept of the spike with real axis (*Z_r_*) at the low frequency [59].

The DC conductivity was calculated easily using Equation (1). Table 1 presents the measured conductivity for all samples. It is obviously seen that the conductivity increases with increasing the plasticizer. Glycerol plasticizer (GP) has been shown to improve DC conductivity for various electrolytes by two orders of magnitude in previous studies. The multi OH moiety structure of glycerol serves as an alternate mechanism for free ions from salt to move in PEs. As a result, GP has a lot of OH groups, which allows it to dissociate more salt while also scarifying the intra and inter-hydrogen bonding in the polymer matrix, resulting in increased amorphous structures, which are essential for the ion transport process [60,61,62].

To compare, the present DC conductivity is quite close to that obtained for PVA:NH_4_SCN:Ce(III)-complex:glycerol composite PE (*σ_dc_* = 2.07 × 10^−3^ S/cm) recorded by Brza et al. [2]. Gel PEs generally possess larger ionic conductivity than SPEs without safety concerns of liquid electrolytes [63]. The ionic conductivity in this work is comparable to that obtained by Zhao et al. [64] who prepared stretchable SCs device composed of highly stretchable gel electrolyte and polypyrrole electrodes materials. The gel PE in their study has shown the DC conductivity of 3.4 × 10^−3^ S cm^−1^ for PVA/H_3_PO_4_. The acceptable level of DC conductivity of the electrolyte is in the range between 10^−3^ to 10^−5^ in order for the electrolyte to be used for application in electrochemical devices and industry [65,66,67].

To investigate the EIS of each electrolyte, it is easy to use the electrical equivalent circuit (EEC) model [68]. From the Nyquist plots and modeling the EEC of the electrolytes, a constant phase element, i.e., *CPE* and the *R_b_* for the carriers are achieved as indicated in the Figure 2a–e inset. The fitting parameters of EEC are indicated in Table 2. The *Z_CPE_* is expressed as follow [31]:

(2) $$Z_{CPE}=\frac{\cos(\pi n/2)}{C\omega^{n}}-j\frac{\sin(\pi n/2)}{C\omega^{n}}$$

where the *CPE* capacitance is symbolized as *C*, the angular frequency is assigned as *ω* and *n* is associated to the imaginary axis deviation of the Nyquist plot. It is established that the Nyquist plot shows that the just the resistive component of the electrolyte is dominated. The polymer can also behave like an insulator and the *CPE* and *R_b_* are connected together in series as revealed in the Figure 2a–e inset. The semicircle disappearance in the electrolyte systems is because most of the anions and cations in the BE transfer to the surface of the electrodes to generate the DL. In this case, the values of *Z_r_* and *Z_i_* of the EEC can mathematically be shown as follow [31]:

(3) $$Z_{r}=R+\frac{\cos(\pi n/2)}{C\omega^{n}}$$

(4) $$Z_{i}=\frac{\sin(\pi n/2)}{C\omega^{n}}$$

In Table 2, *K* is the reciprocal of capacitance at the low frequency region.

As the Nyquist plot plot consists of a spike only, the mobility (*μ*), diffusion coefficient (*D*) and number density (*n*) of ions are determined using the below relations [2]:

The *D* of the ions of each system is determined using the relation:

(5) $$D=D_{{}^{\circ}}\exp\{-0.0297{\left[\ln D_{{}^{\circ}}\right]}^{2}-1.4348\ln D_{{}^{\circ}}-14.504\}$$

where:

(6) $$D_{{}^{\circ}}=\left(\frac{4k^{2}l^{2}}{R_{b}{}^{4}\omega_{\min}{}^{3}}\right)$$

where l is the film thickness and *ω_min_* refers the angular frequency corresponding to the lowest value of *Z_i_*.

The mobility (*µ*) of the ions is determined using Equation (7):

(7) $$\mu =\left(\frac{eD}{K_{b}T}\right)$$

where *k_b_* and *T* refer the Boltzmann constant and the absolute temperature, respectively.

Since conductivity of carriers is determined by:

(8) $$\sigma_{Dc}=ne\mu$$

Thus, the *n* of ions is determined using Equation (8):

Table 3 reveals the *ω*_min_ values and the transport parameter values for each electrolyte system.

On the basis of Table 3, the *D* value was increased when the glycerol increased from 10 to 50 wt.%. As seen in Table 3 *μ* showed the same trend where μ increased. The improvement of *D* and *μ* is associated to the chain flexibility development by the adding of glycerol. When more glycerol was added, the *μ*, *D* and *n* improved which caused enhanced conductivity because the addition of more glycerol dissociated extra salts to cations and anions, thus improving the *n* of carriers [2]. Glycerol reduces the attraction force between the anions and cations of the salt [69]. Hence, a higher number of ammonium ions (*n_i_*) is generated by NH_4_I to the polymer. It was documented that [69] the dielectric constant of electrolytes increased with decreasing frequency and thus the capacitance would increase. The capacitance is also increased with increasing the glycerol concentration. The plasticizer to the electrolyte increased the number density of free ions (see Table 3), and hence increased the dielectric constant value as mentioned at the next section.

**Figure 3:**
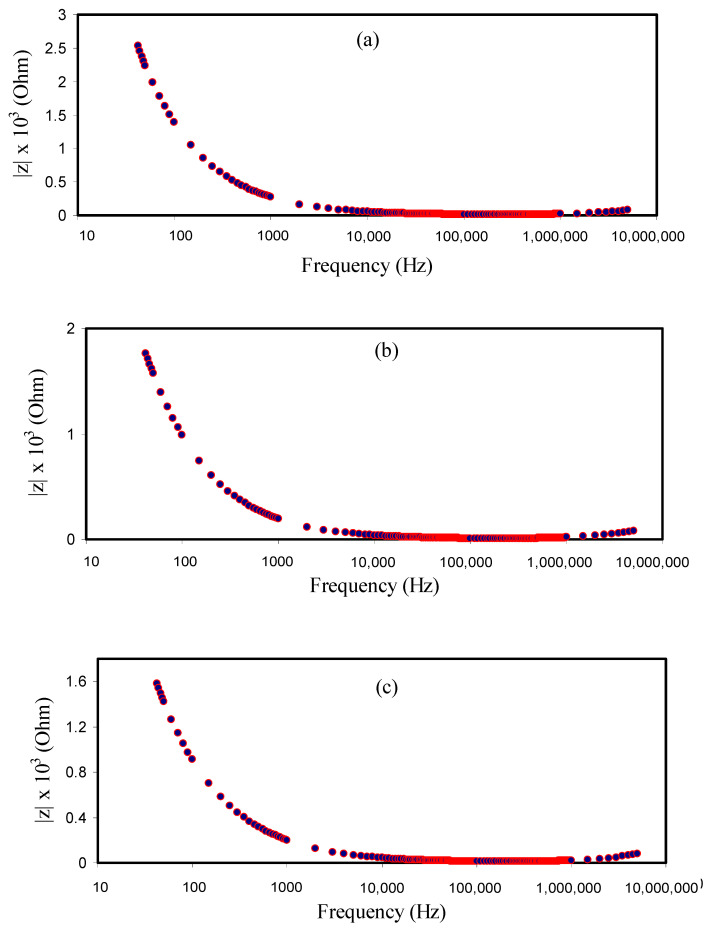

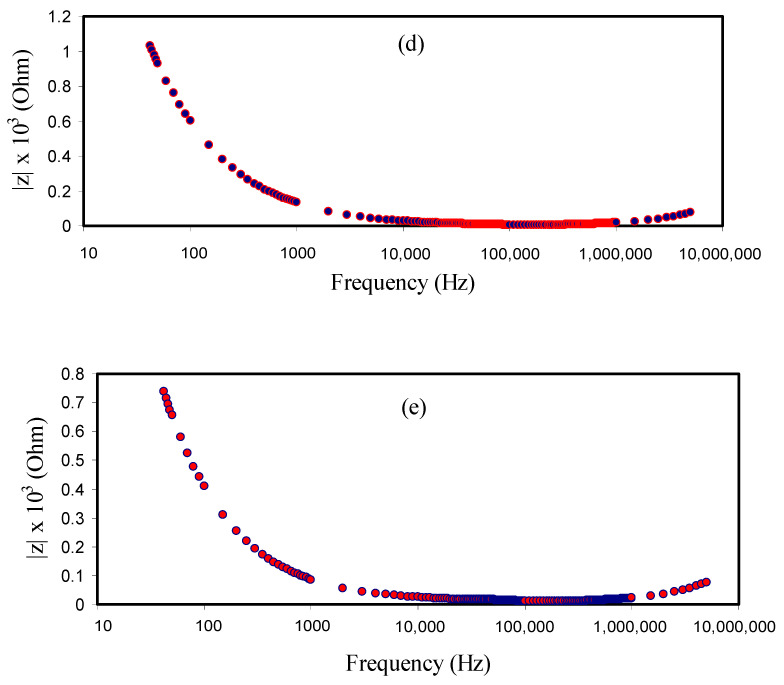
Bode plots for (**a**) MCPVI11, (**b**) MCPVI2, (**c**) MCPVI3, (**d**) MCPVI4 and (**e**) MCPVI5 electrolyte films.

It is observed in Figure 3a that the MCPVI1 film has the largest charge transfer resistance (R_ct_ = 2.56 Ω). Obviously, with rising concentration of glycerol, as revealed in Figure 2b–e, the R_ct_ decreased to 0.76 Ω. The dispersion region at the low-frequency region in the Bode plots is ascribed to the diffusion of ions phenomenon and the high-frequency region is attributed to the R_ct_ [70]. In Figure 2 and Figure 3, it is shown that the MCPVI5 film has the minimum R_ct_ and, thus, a higher DC conductivity was obtained. Thus, the Bode plot agreed with the results achieved from the EIS plots. From a physics viewpoint, it is vital to fabricate PE with higher conductivity, while it is vital for the films to show a low R_ct_ from a chemistry viewpoint [70].

### 3.2. Dielectric Properties

It has been emphasized that understanding and recognizing charge carrier transport mechanisms in electrolytes can be achieved via studying dielectric properties [71,72]. The dielectric loss (ε_i_) and dielectric constant (ε_r_) are frequency dependents that can be calculated using the equations as reported in reference [73].

**Figure 4:**
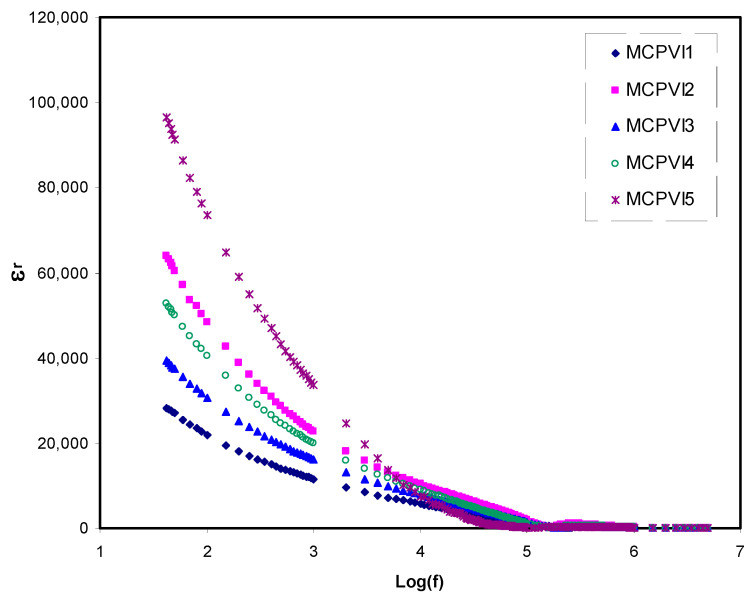
Dielectric constant (ε′) spectra versus frequency for the prepared samples.

**Figure 5:**
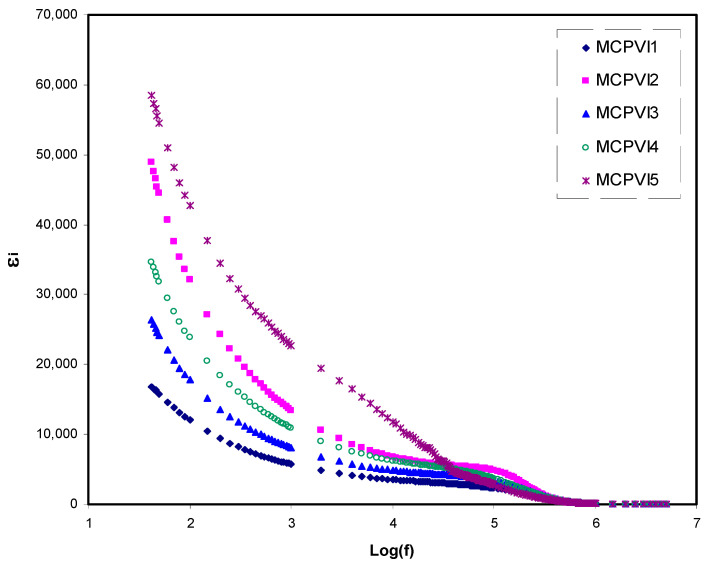
Dielectric loss (ε″) spectra versus frequency for the prepared samples.

The ε_r_ and ε_i_ parameters versus frequency upon quantity of plasticizer are shown in Figure 4 and Figure 5, respectively, at ambient temperature. The dielectric response exhibits a dispersion region that declined with rising frequency in all samples.

This can be explained on the basis of providing adequate time for charge carriers and dipoles to alter the direction when an electric field is applied at low frequency. As a consequence, building up of large number of charge carrier occurs at the interfacial region accompanied with electrode polarization. This phenomenon results in suppression of dielectric properties at high frequency range [74]. It is also noted that the space-charge disappears at the high-frequency region as a result of prohibition of dipole moment and charge carrier to flow at high periodic reversal form of the applied external AC electric field. Moreover, under this condition there is no ion moving from BE into diffuse layer at the interfacial region. Thus, the dielectric parameters (ε_r_ and ε_i_) decrease gradually as the frequency increases [75,76].

This study has shown that dielectric properties of the PE are highest with incorporation of 50 wt.% of glycerol. This indicates the existence of a large number of charge carrier and thus relatively high conductivity [77].

### 3.3. Transference Number Measurement (TNM) and Linear Sweep Voltammetry (LSV) Studies

**Figure 6:**
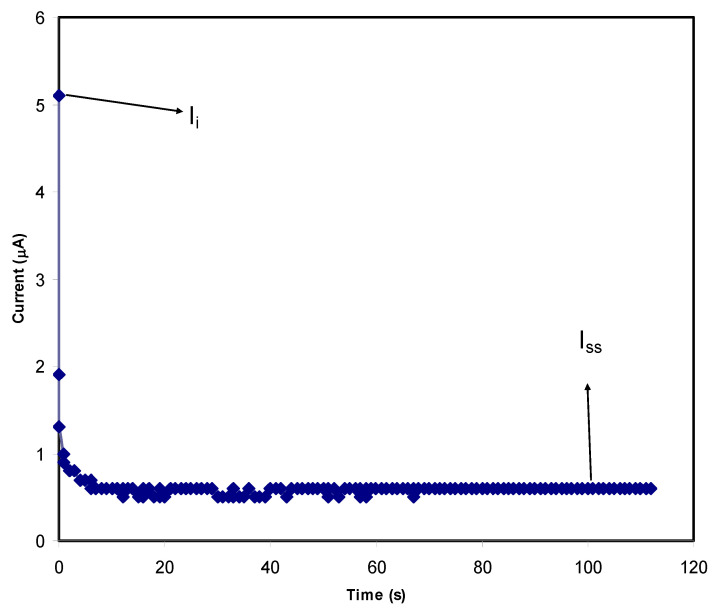
TNM plot for PVA:MC:NH_4_I electrolyte with 40 wt.% glycerol.

**Figure 7:**
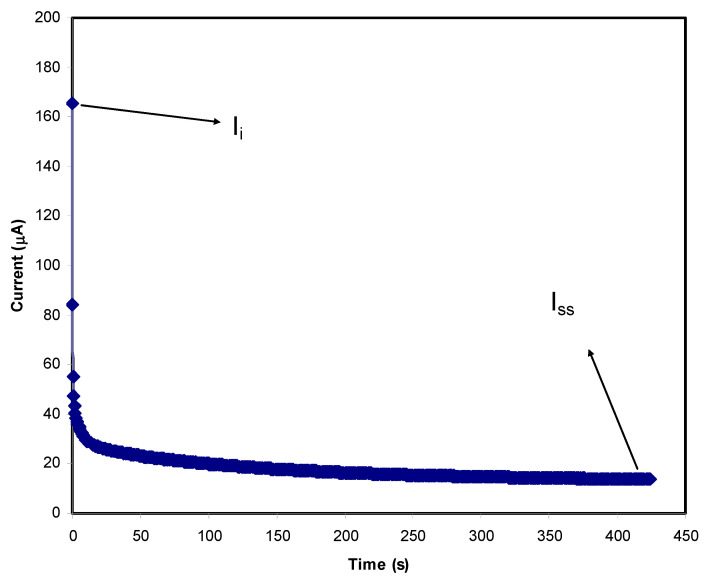
The TNM plot for PVA:MC:NH_4_I electrolyte with 50 wt.% glycerol.

The suitability of a PE for the application of a common EDLC function can be identified through both TNM and LSV analyses [50]. There are two types of charge carriers present in a typical PE system which are ions and electrons, and the major conducting components in an electrolyte will be revealed by these studies. Figure 6 and Figure 7 show the TNM plots for the PVA:MC:NH_4_I electrolyte with the addition of 40 wt.% and 50 wt.% glycerol, respectively.

The polarization processes in Figure 6 and Figure 7 show that the initial current (*I_i_*) for the PVA:MC:NH_4_I electrolyte with 50 wt.% glycerol is higher than the electrolyte with 40 wt.% glycerol concentration. This difference exhibits the effect of plasticizer to the amount of conduction species in the electrolyte. However, both electrolytes achieved high initial current which is contributed by ions and electrons. Then, a drastic current drop is observed for both plots due to the blocking of ions caused by the characteristics of the stainless steel electrode. The electrode only allows the electrons to move. The current decay region is where the ions’ diffusion process is equivalent to ions’ drifting within a short time period. The polarization happens for a long time period at the steady state because of the diffusion layer development on the interface’s region. Additionally, this phenomenon is also dealing with the high resistance of the formation of the passive layer by ions, thus the current flow is totally caused by the electrons without the involvement of ions [50]. Based on the plots, the electrolyte with a high concentration of glycerol is observed to require longer time for the current to reach steady state (*I_SS_*) as the low concentration requires less time. By using the Equations (9) and (10), the value of the electronic (*t_elec_*) and ionic (*t_ion_*) transference numbers can be determined and tabulated in Table 4.

(9) $$t_{ion}=\frac{I_{i}-I_{ss}}{I_{i}}$$

(10) $$t_{elec}=1-t_{ion}$$

According to the Table 4, it was discovered that both electrolytes achieved high *t_ion_* values which led to an explanation that both systems are dominantly contributed by the ions to serve as the conducting element [78]. The addition of 50 wt.% glycerol into PVA:MC:NH_4_I electrolyte caused the *t_ion_* to be higher at 0.917 with *t_elec_* value of 0.083 compared to 40 wt.% glycerol electrolyte. The result obtained from this analysis verifies that the addition of 50 wt.% glycerol into PVA:MC:NH_4_I electrolyte can produce a highly ion contributing electrolyte that is fits to be applied in EDLC where the electromotive force will be occurred between carbon electrodes and ions [79,80]. High *t_ion_* value was also reported by Shukur et al. [42] for the plasticized electrolyte based on the chitosan-NH_4_Br polymer blend and the electrolyte showed a promising performance in the EDLC application.

**Figure 8:**
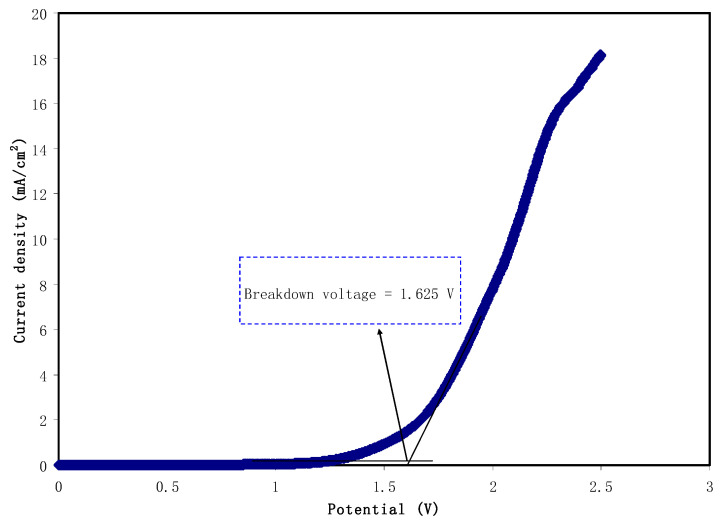
The LSV of the highest conducting PVA:MC:NH_4_I:Gly electrolyte at room temperature.

The working potential range is one of the critical characteristics that needs to be verified because it will determine the breakdown voltage of the electrolyte. LSV analysis was carried out at room temperature for the PVA:MC:NH_4_I:Gly electrolyte with the highest conductivity value as illustrated in Figure 8. Based on the plot, it was observed that no obvious J was recorded through the applied voltage from 0 to 1.625 V. According to Sampathkumar et al. [81], no electrochemical reaction occurs within the electrolyte at this region. However, when the voltage increased more than 1.65 V, the J started to gradually increase which correlated to the breakdown point of electrolyte and also indicated the occurrence of electrochemical reaction [82]. A comparable LSV result was reported by Asnawi et al. [83] for the plasticized chitosan-NH_4_F electrolyte system. For the fabrication of energy devices, the minimum breakdown voltage is 1.0 V and the electrolyte in this work shows a promising stability, hence it is suitable to be applied in a common EDLC application [84]. Bockenfeld et al. [85] utilized protic ionic liquid electrolyte for a lithium ion battery. They showed that the electrolyte 0.5 M lithium nitrate (LiNO_3_) in propylene carbonate (PC)-pyrrolidinium nitrate (PYRNO_3_) showed the total potential stability of 2.65 V. G A M Ali et al. [86] prepared carbon nanospheres (NSs) by seeds of lablab purpureus and they revealed that the carbon NSs materials are valuable in applications of SCs electrode. The authors indicated that the practical symmetrical SCs has a good electrochemical behavior under potentials window up to 1.7 V.

### 3.4. Characterization of EDLC

**Figure 9:**
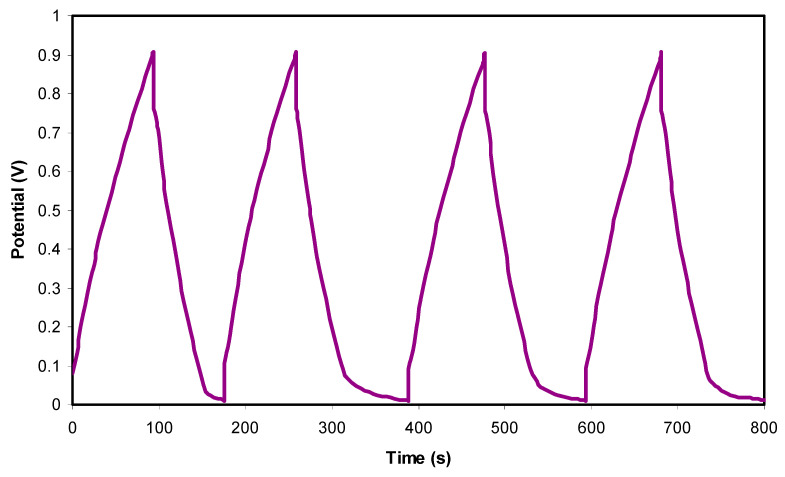
Galvanostatic charge-discharge plot of the fabricated EDLC for selected cycle.

The Galvanostatic charge-discharge (GCD) measurement had been used to evaluate the performance of EDLC as well as revealing few significant properties such as equivalent series resistance and specific capacitance. The GCD plot of the fabricated EDLC using the highest conducting PVA:MC:NH_4_I:Gly electrolyte is depicted in Figure 9 for selected cycles. Additionally, the linear discharge slope indicates the capacitive behavior of the EDLC that might be caused by the ionic DL at the electrode/electrolyte region [43]. A small voltage drop, *V_drop_* is obtained by the GCD. As stated by Farah et al. [87], the occurrence of *V_drop_*—also called ohmic loss—is due to the internal resistance such as electrolyte’s bulk resistance and charge transfer resistance which are produced between the electrode and electrolyte interface area within the EDLC. As the cycle number is increased, the *V_drop_* value tends to increase because of the mobile ions’ reduction due to their accumulation and migration at the DL region [88].

The internal resistance, also known as equivalent series resistance (*ESR*) that exists due to the presents of *V_drop_* can be expressed using the Equation (11):

(11) $$ESR=\frac{V_{drop}}{i_{}}$$

where *i* represents the operation current.

**Figure 10:**
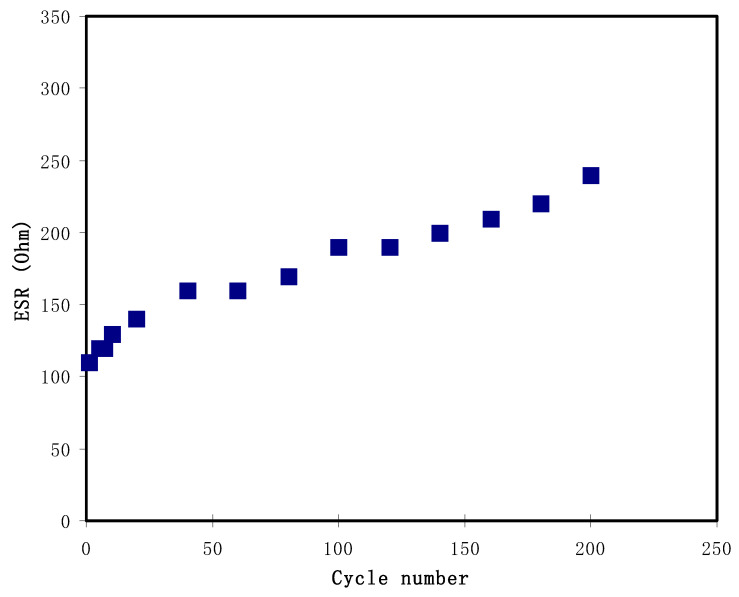
The equivalent series resistance, *ESR* from GCD plot for 200 cycles.

The calculated value of *ESR* for 200 cycles can be observed in Figure 10. The first cycle of EDLC shows the *ESR* value of 110 Ohm and then a gradually increment of *ESR* can be noticed throughout the cycles; 160 Ohm at the 40th cycle and 240 Ohm at the final cycle. Various factors have been reported to cause the existence of *ESR*, where the first one is due to the current collectors used for the fabrication which are aluminum foil. Next, the rapid charge-discharge within the electrolyte will cause the free ions to recombine and lead to the decrement of ionic conductivity. Kang et al. [89] agreed that *ESR* was strongly related to the conductivity of an electrolyte. Additionally, the DL charge is established by the activities at the space between electrode/electrolyte which are movements of electrons and ions [90]. Wang et al. [91] studied the EDLC using starch-lithium acetate system and exhibited a similar trend as in this work where *ESR* increased while the *C_s_* values were maintained at an approximate constant value.

**Figure 11:**
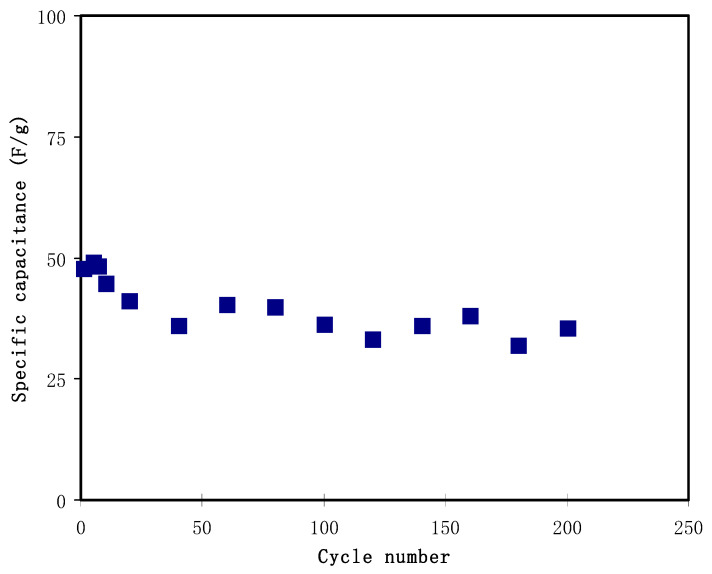
The specific capacitance, *C_s_* from GCD plot for 200 cycles.

The specific capacitance (*C_s_*) had been measured using the equations as reported in references [92,93,94]. The mass of activated carbon used for the calculation is 2.43 × 10^−3^ g. The determined *C_s_* values are plotted in the Figure 11 for 200 cycles. The calculated *C_s_* value is achieved by the fabricated EDLC is 47.85 F/g at the first cycle. Then, the *C_s_* value lowers to 36.10 F/g when the cycle number increases to 40 and the values then start to stay at approximately 35.48 F/g until the 200th cycle. The reduction of *C_s_* values might be caused by the weak contact at electrode/electrolyte region [95,96]. The almost constant values achieved at the end of cycle number are because the electrolyte is slowly polarized, and the accumulation of charge occurred on both electrodes; this whole process is called the stabilization of ion polarization [66]. Azli et al. [97] has reported comparable *C_s_* values calculated based on GCD plot by using similar activated carbon electrodes. G A M Ali et al. [98] synthesized porous nanocarbons (NCs) using bio-waste oil palm leaf. The NCs indicated good SCs property. They reported a high specific capacitance of 368 F/g at 0.06 A/g in 5 M KOH. Low resistance values were achieved in their study indicating the porous NCs availability as precursor in the creation of SCs electrodes. In another study, G A M Ali et al. [86] created carbon NSs by lablab purpureus seed. The authors documented that the carbon NSs were useful for electrode applications in SCs. The specific capacitances in their study were determined to be 300, 265 and 175 F g^−1^ in 5 M KOH electrolyte for carbon NSs prepared at 800, 700 and 500 °C, respectively.

Minakshi et al. [99] showed that the ES of sodium and lithium ions from aqueous solution in binary metal oxide (BMO) was important for uses of renewable ES. They mentioned the BMO as a novel electrode material for SCs. They showed that the binary metal oxide of CaMoO_4_ in salt-in-water NaOH at 0.5 A g^−1^ had a specific capacitance of 206 F/g. Biswal et al. [100] prepared hierarchical porous cobalt (CO)–nickel (Ni)–iron (Fe) ternary oxide heterostructure (HS) as an electrode material for application in hybrid capacitors due to their large redox potentials. The authors examined role of concentration of Fe in ternary oxide HS as ES material on the hybrid device. They showed that the increasing Fe concentration in the metal oxide reduced the specific capacitance from 440 F/g to 272 F/g, representing a large difference in the observed redox mechanisms.

**Figure 12:**
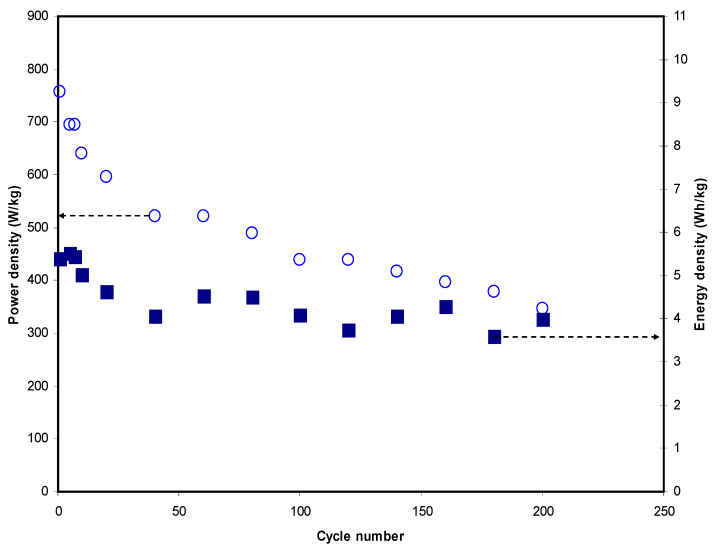
The EDLC energy density (*E_d_*) and power density (*P_d_*) versus cycle number for 200 cycles.

It is also important to identify the energy density (*E_d_*) and power density (*P_d_*) of the fabricated EDLC which has been calculated using the equations as reported in references [15]. Figure 12 shows the *E_d_* and *P_d_* of the fabricated EDLC for 200 cycles. The *E_d_* plot is noticed to be in a good agreement with the *C_s_* plot. The calculated *E_d_* for the first cycle of the EDLC was 5.38 Wh/kg and dropped to 4.63 Wh/kg at cycle number 20. The values were found to stabilize between 4.54 to 3.59 Wh/kg from the 60th cycle towards the end. These stabilize readings were caused by the amount of energy required for the movements of ions from the BE towards the electrode’s surface which was almost similar [101]. Furthermore, the *P_d_* plot is also depicted in Figure 12. Along the 200 cycles of charge-discharge, *P_d_* values of the EDLC experienced a step-by-step drop from 757.58 to 347.22 W/kg. The relatively high *E_d_* and *P_d_* results obtained in this work were related to the high porosity and large surface area of the electrode material for the fabrication of EDLC [102]. The decrement of the EDLC characteristic parameters of energy and power densities at high cycle number was generally caused by the recombination of ions which was due to the increase of internal resistance during the rapid charge-discharge process [103].

Gomaa A. M. Ali et al. [58] prepared activated carbon using palm kernel shell (ACPKS) and impregnated it with CaO by eggshell (CaO/ACPKS) as SCs electrodes materials. They showed that the practical symmetrical supercapacitor of CaO/ACPKS had high *E_d_* of 27.9 W h/kg at a *P_d_* of 85.7 W kg^−1^. Gomaa A. M. Ali et al. [104] in another study synthesized grapheme-nanosheets (GNSs) as SCs electrodes. They stated that the GNSs had a specific capacitance of 140 F g^−1^ at 0.05 A g^−1^, and the *E_d_* ranged from 5 to 4 Wh/kg and *P_d_* ranged from 135 to 2818 W kg^−1^.Minakshi et al. [105] synthesized a hybrid ESD by sustainable electrode materials. They documented that the hybrid device generated an *E_d_* of 35 Wh/kg and a *P_d_* of 420 W/kg with long term cyclability of 98% retention of initial capacitance after 1000 cycles. They showed that the new hybrid ESD, which was prepared from bio-waste materials such as eggshells in combination with cheap and available mixed metal oxides (NiO/Co_3_O_4_), had more applications for numerous energy intensive.

## 4. Conclusions

Summarily, preparation of PVA/MC-NH_4_I PEs was carried out with plasticization using various quantities of glycerol using solution casting methodology. It was concluded that plasticization of PVA/MC-NH_4_I PEs gave a relatively high value of DC conductivity, which is decisive property to be applicable in EDLC. It was revealed that when the glycerol increased, the mobility, diffusion coefficient and number density of ions gradually increased. It was also confirmed that the relatively high dielectric properties could be achieved when carrier density increased. The transference number measurement (TNM) study presents the electrolyte with 50 wt.%, while glycerol has higher *t_ion_* value than electrolyte with 40 wt.% glycerol which is 0.917 and 0.882, respectively. The high *t_ion_* compared to *t_elec_* proves the domination of ion as the conducting species in the electrolyte. From the LSV response, the breakdown voltage of the electrolyte is determined and found to be 1.625 V. From GCD plot, the calculated *C_s_* values are found to drop from 47.85 F/g to 35.48 F/g throughout the 200 cycles. The increment of internal resistance is shown by *ESR* plot. The EDLC achieved energy density of 5.38 Wh/kg at the 1st cycle and stabilized between 4.54 to 3.59 Wh/kg from the 60th cycle towards the end. The power density values experienced a step-by-step drop from 757.58 to 347.22 W/kg.

## Figures and Tables

**Table 1:** DC conductivity for plasticized electrolytes at room temperature.

Designation	Conductivity (S·cm^−1^)
MCPVI1	4.12 × 10^−4^
MCPVI2	8.21 × 10^−4^
MCPVI3	1.01 × 10^−3^
MCPVI4	1.29 × 10^−3^
MCPVI5	1.80 × 10^−3^

**Table 2:** The EEC fitting parameters for plasticized electrolyte systems at room temperature.

Sample	*n* (rad)	*K* (F^−1^)	*R_b_* (Ω)	*CPE* (F)
MCPVI1	0.706	6.13 × 10^4^	(0.16 ± 0.060) × 10^2^	(1.63 ± 0.58) × 10^−5^
MCPVI2	0.63	6.125 × 10^4^	(0.14 ± 0.061) × 10^2^	(1.633 ± 0.60) × 10^−5^
MCPVI3	0.658	6.12 × 10^4^	(0.13 ± 0.062) × 10^2^	(1.634 ± 0.61) × 10^−5^
MCPVI4	0.636	6.11 × 10^4^	(0.12 ± 0.061) × 10^2^	(1.64 ± 0.60) × 10^−5^
MCPVI5	0.674	3.61 × 10^4^	(0.105 ± 0.059) × 10^2^	(2.77 ± 0.68) × 10^−5^

**Table 3:** The *ω*, *µ*, *D* and *n* values at room temperature.

Sample	*ω* (rad s^−1^)	*D* (cm^2^ s^−1^)	*µ* (cm^2^ V^−1^ s)	*n* (cm^−3^)
MCPVI1	2.20 × 10^5^	1.59 × 10^−6^	6.20 × 10^−5^	4.15 × 10^19^
MCPVI2	7.54 × 10^4^	2.39 × 10^−6^	9.32 × 10^−5^	5.50 × 10^19^
MCPVI3	1.63 × 10^5^	2.42 × 10^−6^	9.45 × 10^−5^	6.67 × 10^19^
MCPVI4	1.26 × 10^5^	2.43 × 10^−6^	9.48 × 10^−5^	8.49 × 10^19^
MCPVI5	1.30 × 10^5^	2.44 × 10^−6^	9.51 × 10^−5^	1.18 × 10^20^

**Table 4:** Transference number for PVA:MC:NH_4_I:Gly electrolytes.

Glycerol (wt.%)	*t_elec_*	*t_ion_*
40	0.118	0.882
50	0.083	0.917

## Data Availability

Data sharing not applicable.
